# Farnesoid X Receptor Agonist INT-787 Inhibits Hepatic Mitochondrial Dysfunction in a Diet-Induced ob/ob Mouse Model of MASH

**DOI:** 10.3390/ijms262211023

**Published:** 2025-11-14

**Authors:** Laura Giuseppina Di Pasqua, Giuseppina Palladini, Anna Cleta Croce, Gloria Milanesi, Margherita Cavallo, Luciano Adorini, Andrea Ferrigno, Mariapia Vairetti

**Affiliations:** 1 Department of Internal Medicine and Therapeutics, University of Pavia, Via Ferrata 9, 27100 Pavia, Italy; lauragiuseppina.dipasqua@unipv.it (L.G.D.P.); giuseppina.palladini@unipv.it (G.P.); mariapia.vairetti@unipv.it (M.V.); 2 Internal Medicine and Emergency Medicine Unit, Fondazione IRCCS Policlinico San Matteo, 27100 Pavia, Italy; 3 Institute of Molecular Genetics, Italian National Research Council (CNR), Via Abbiategrasso 207, 27100 Pavia, Italy; annacleta.croce@igm.cnr.it; 4 Department of Biology & Biotechnology, University of Pavia, Via Ferrata 9, 27100 Pavia, Italy; gloria.milanesi@unipv.it (G.M.); margherita.cavallo01@universitadipavia.it (M.C.); 5 Intercept Pharmaceuticals, San Diego, CA 92121, USA; ladorini@interceptpharma.com

**Keywords:** FXR, INT-787, MASH, mitochondria, MIC19

## Abstract

This study evaluated the protective role of farnesoid-X-receptor (FXR) agonist INT-787 in the control of mitochondrial changes using a metabolic dysfunction-associated steatohepatitis (MASH) model. Lep-ob/ob mice were fed a control diet (CD) for 21 weeks (wks), or a high-fat diet (HFD) for 9 or 21 wks; in the 21 wk HFD groups, INT-787 (30 mg/kg/day) dosed via HFD admixture was added. The hepatic ATP, ROS, GSH and MIC19, which stabilizes the structure of inner mitochondrial membrane (IMM), were quantified. Transmission electron microscopy (TEM) analysis was also performed. INT-787 increased hepatic ATP, which was downregulated after HFD 9 and 21 wks. Hepatic ROS increased and GSH decreased after 21 wks and were recovered by INT-787. MIC19 mRNA level decreased after HFD 21 wks, and it was completely restored after INT-787 administration. TEM analysis showed that INT-787 reverted the mitochondrial alterations as documented by restored mitochondrial length, number of mitochondrial cristae junctions (CJs), and distance between endoplasmic reticulum (ER) and outer mitochondrial membrane (OMM) when compared with HFD groups. These results underline the involvement of the FXR pathway in the control of mitochondrial damage, thus revealing a previously undiscovered mechanism mediated by FXR activation: the upregulation of IMM protein MIC19, which is essential for maintaining cristae integrity and mitochondrial function.

## 1. Introduction

The liver, responsible for carbohydrate, protein, and lipid metabolism, is one of the richest organs in number and density of mitochondria, required for multiple metabolic pathways including β-oxidation, tricarboxylic acid (TCA) cycle, ketogenesis, respiratory activity, and ATP synthesis [1].

Mitochondria, critical organelles involved in energy production, play a role in the electron transport chain linked to the production of reactive oxygen species (ROS) [2]. Recent studies have highlighted the importance of mitochondrial processes in the balance between liver function and disease progression [2]. When these processes are disrupted, mitochondrial alterations cause several cellular stresses, such as increased ROS production and the development of metabolic diseases in the liver [3].

According to the revised terminology, the acronym NAFLD has been replaced by MAFLD, to indicate “metabolic dysfunction associated fatty liver disease” [4]. MAFLD identifies patients with significant hepatic fibrosis more accurately than NAFLD. Recently, the results of a Delphi consensus statement on new fatty liver disease nomenclature chose metabolic dysfunction—associated steatotic liver disease (MASLD) as the replacement term for NAFLD and metabolic dysfunction—associated steatohepatitis (MASH) as the replacement term for NASH [5].

MASH pathogenesis is induced in response to fat accumulation in hepatocytes, coupled with mitochondrial dysfunction, including alterations in enzyme activities, protein expression, and signaling networks [6]. These processes are tightly coupled with mitochondrial quality control, hepatocyte cell death and inflammatory responses [7].

Previous studies have shown that mitochondrial dysfunction influences the disease; thus, it is crucial to understand how mitochondria function in MASLD/MASH livers in order to prevent and/or to reverse this condition [8,9].

Mitochondrial cristae host functional respiratory complexes that improve the efficiency of ATP production [10]. The formation and dynamics of these structures is regulated by different components, including the mitochondrial contact site and cristae organizing system (MICOS) protein complex [11]. MIC19 is one of the core subunits of MICOS complex, a multi-subunit complex of proteins, which stabilizes the structure of inner mitochondrial membrane (IMM). The inner boundary membrane (IBM) and the cristae membranes represent the IMM. The cristae junctions (CJs) are the connections between IBM and cristae membranes. Mitochondrial cristae and number of CJs are essential to preserve mitochondrial morphology, structure, and function [12]. Recently, Sohn et al. (2023) evaluated the hepatic cristae formation upon nutritional changes and documented how hepatic MIC19 influences cristae remodeling allowing the maintenance of systemic energy balance [13].

Emerging evidence indicates that not only mitochondrial dysfunction but also endoplasmic reticulum (ER) stress are involved in the initiation and progression of MASLD [14]. A thorough investigation of the cellular mechanisms involved in ER and mitochondrial functions may provide different options to treat MASLD or MASH by targeting ER stress and mitochondrial dysfunction.

We previously demonstrated, using a validated mouse model of Amylin Liver NASH (AMLN) diet-induced MASH [15], the positive effects of FXR agonist INT-787 against MASH-associated liver injury, lipid accumulation, inflammation, and fibrogenesis [16]. The objective of the present study was to further assess whether INT-787 could exert restorative effects, particularly on mitochondrial dysfunction, in the context of prolonged disease progression.

INT-787, a novel Obeticholic acid (OCA) derivative hydroxylated at position C11β, results in a compound equipotent to OCA at FXR (EC50 = 0.14 μmol/L). This modification makes it highly selective for FXR, with no TGR5 agonistic activity, reducing off-target effects [17]. The presence of an additional hydroxyl group imparts unique physicochemical characteristics, aligning it more closely with hydrophilic bile acids like ursodeoxycholic acid [17]. INT-787 is a biologically active and well-tolerated compound, showing rapid absorption and dose-dependent exposure in healthy volunteers [18]. Mechanistically, INT-787 activates FXR signaling more broadly across hepatic pathways and exerts a stronger impact on stellate cell activation, fibrogenesis, and inflammation than OCA [16].

Based on the above considerations, we have evaluated the mitochondrial alterations in a diet-induced ob/ob mouse model and the protective role of INT-787 in the control of mitochondrial dysfunction by assessing hepatic ATP, ROS, MIC19, as well as mitochondrial morphology by transmission electron microscopy (TEM) analysis. Specifically, we now emphasize how MIC19’s role in mitochondrial integrity offers a mechanistic link between FXR signaling and mitochondrial dysfunction in MASH—an area that has not been fully explored in previous FXR agonist studies.

We demonstrate that INT-787 administration reverts the decrease in MIC19 mRNA and improves mitochondrial ultrastructure abnormalities, including the decrease in mitochondrial length, the loss of number of CJs and the increase in distance between ER and outer mitochondrial membrane (OMM).

## 2. Results

**Figure 1:**
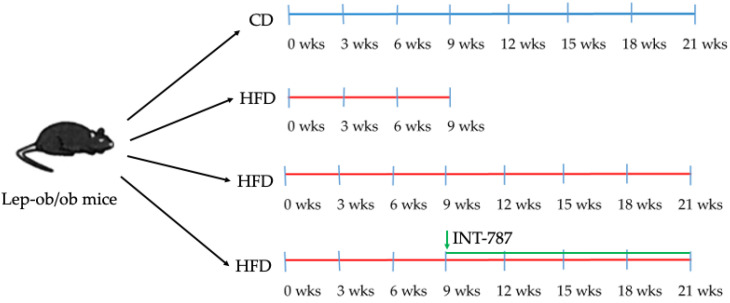
Summary of experimental groups and treatments. Lep-ob/ob mice on HFD for 9 wks were randomized to treatment with INT-787 (30 mg/kg) for a further 12 wks. CD, control diet; HFD, high-fat diet. Mice groups: CD 21 wks (*n* = 7), HFD 9 wks (*n* = 6), HFD 21 wks (*n* = 10) and HFD 21 wks treated with INT-787 30 mg/kg for 12 wks (*n* = 10).

The present study evaluates the protective role of FXR agonist INT-787 in a MASH experimental model. Lep-ob/ob mice received a control diet (CD) for 21 weeks (wks), or a high-fat diet (HFD) for 9 or 21 wks; in the 21 wk HFD groups, INT-787 (30 mg/kg/day) dosed via HFD admixture was added during the final 12 wks as summarized in Figure 1. INT-787 dose was determined according to previous studies [16,19].

An increased liver weight was found in HFD-treated mice at 9 and 21 wks as compared with CD mice (Table 1). In INT-787-treated mice a reduction in liver weight to values similar to the CD group has been observed (Table 1).

Serum ALT increased in HFD groups and peaked after 9 wks (Table 1). INT-787 administration significantly reduced serum levels of ALT compared with all groups considered (Table 1). Alkaline Phosphatase (ALP) increased after HFD 9 wks as compared with other groups, but the administration of INT-787 was ineffective in reducing ALP values (Table 1). INT-787 treatment reduced serum bilirubin compared with HFD 9 and 21 wks (Table 1). A marked reduction in serum bile acids was found after INT-787 administration compared with all groups considered (Table 1). Serum cholesterol increased in mice after HFD 9 and 21 wks compared with CD group; INT-787 administration markedly counteracted these increases to values observed in CD group (Table 1). No difference in serum triglycerides occurred in all groups considered (Table 1).

**Figure 2:**
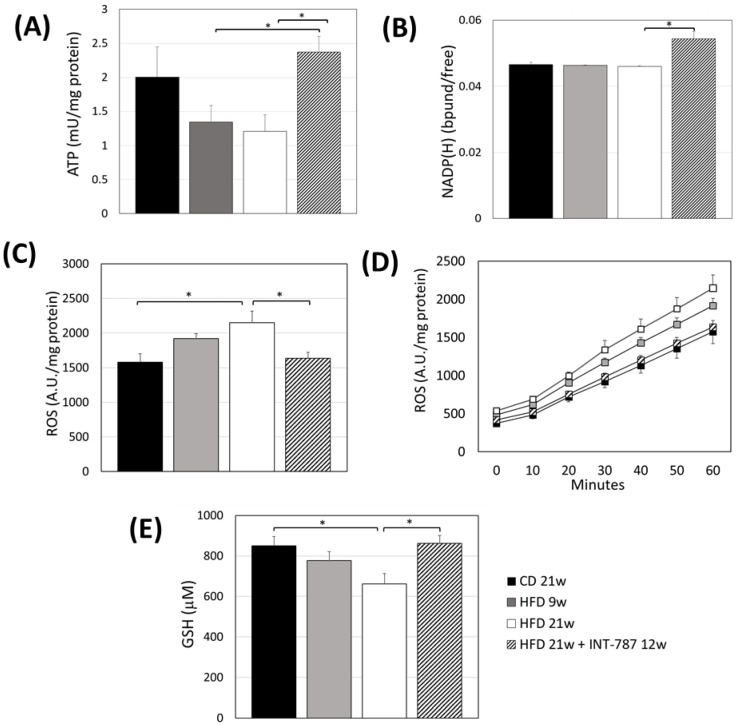
Effect of INT-787 on hepatic ATP (**A**), NADP(H) (**B**), ROS (**C**,**D**), and GSH (**E**) in a diet-induced ob/ob mouse model of MASH. Mice groups: CD 21 wks (*n* = 7), HFD 9 wks (*n* = 6), HFD 21 wks (*n* = 10) and HFD 21 wks treated with INT-787 30 mg/kg for 12 wks (*n* = 10). Data are shown as mean  ±  SEM; * *p* ≤ 0.05. ANOVA followed by Tukey post hoc multiple comparisons were used for the analysis of ROS and GSH. When data distribution was not normal, Kruskal–Wallis and Dunn’s tests were used: ATP and NADP(H).

A reduction in hepatic ATP content was found in HFD-treated mice after 9 and 21 wks (*p* ≤ 0.05), confirming that the HFD administration promotes mitochondrial dysfunction (Figure 2A). Higher levels of hepatic ATP were documented in INT-787 group when compared with HFD groups (Figure 2A).

We previously reported the use of liver autofluorescence (AF) of NADPH bound/free ratio and its correlation with ATP as tissue biomarker of energy metabolism [20]. The present study shows a recovery by INT-787 treatment in NADPH bound/free ratio compared with all groups considered (*p* ≤ 0.05) (Figure 2B).

An increase in hepatic ROS formation was found at HFD 21 wks compared with CD mice (*p* ≤ 0.05) (Figure 2C,D). Conversely, lower ROS formation was observed in INT-787 group compared with HFD 21 wks group (Figure 2C,D).

Lower levels of GSH were found in mice fed a HFD 21 wks compared to those on a CD group (*p* ≤ 0.05) (Figure 2E). A recovery in GSH levels was observed following INT-787 administration, with values comparable to those found in the CD group (Figure 2E).

**Figure 3:**
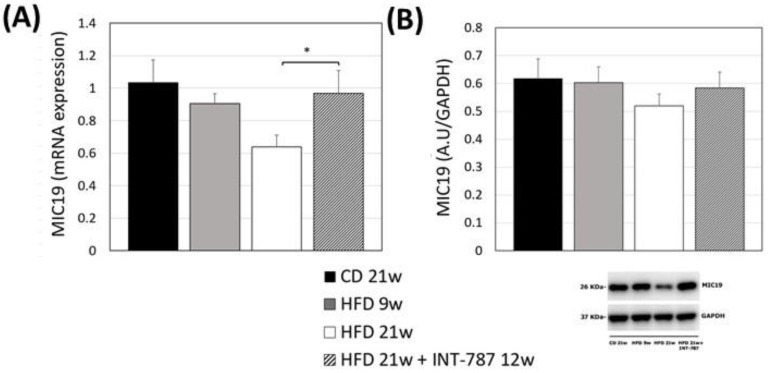
Effect of INT-787 on hepatic MIC19 mRNA (**A**) and protein (**B**) in a diet-induced ob/ob mouse model of MASH. Mice groups: CD 21 wks (*n* = 7), HFD 9 wks (*n* = 6), HFD 21 wks (*n* = 10) and HFD 21 wks treated with INT-787 30 mg/kg for 12 wks (*n* = 10). Data are shown as mean  ±  SEM; * *p* ≤ 0.05. ANOVA followed by Tukey post hoc multiple comparisons were used.

MIC19 depletion has been reported to cause mitochondrial phospholipids disorders leading to structural abnormalities and mitochondrial stress [12]. We performed the analysis of MIC19 mRNA, demonstrating a significant decrease in its expression in mouse liver after 21 wks HFD (*p* ≤ 0.05) (Figure 3A). INT-787 administration reverted this trend increasing the levels of MIC19 mRNA to values comparable with those observed in CD mice (Figure 3A). Although not significant, the same trend was noted for MIC19 protein (Figure 3B).

**Figure 4:**
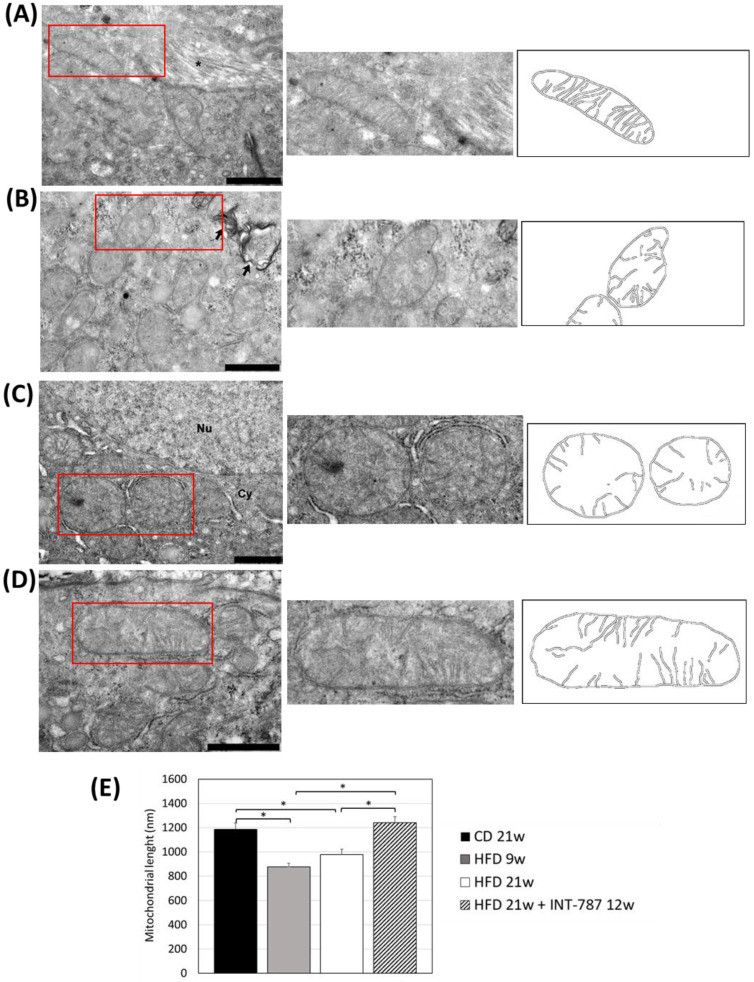
In vivo treatment with INT-787 restores hepatic mitochondrial length in HFD-fed ob/ob mouse: CD 21 wks (**A**), HFD 9 wks (**B**), HFD 21 wks (**C**) and HFD 21 wks treated with INT-787 30 mg/kg for 12 wks (**D**). Representative TEM pictures: *n* = 3 independent experiments. Liver magnification: bar 1 µm; the selected area (red boxes) was subsequently enlarged (2× zoom), on the right, and analyzed using Image J (FIJI 1.54p) Legend: *: Collagen fibers, arrows: phagosomes; Nu: nucleus; Cy: cytoplasm. (**E**) Image J quantification (nm). Data are shown as mean  ±  SEM; * *p* < 0.0001. Kruskal–Wallis and Dunn’s tests were used.

**Figure 5:**
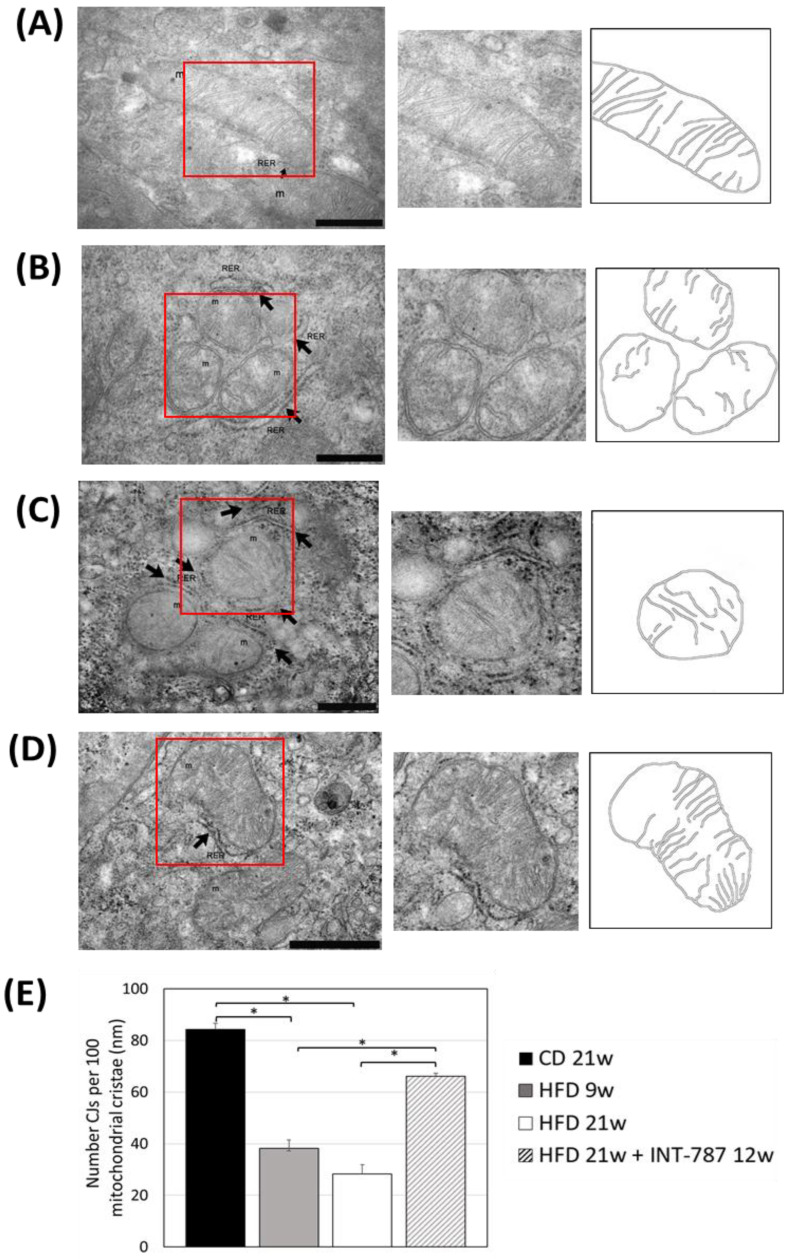
In vivo treatment with INT-787 restores the number of cristae junctions (CJs) in HFD-fed ob/ob mouse model of MASH. Representative TEM pictures: *n* = 3 independent experiments. Liver magnification: (**A**–**C**), bar 500 nm; (**D**), bar 1 µm; the selected area (red boxes) was subsequently enlarged (2× zoom), on the right, and analyzed using Image J (FIJI 1.54p). Legend: m: mitochondria, RER: rough endoplasmic reticulum, arrows: RER. (**E**) Number of cristae junctions (CJs) per 100 mitochondrial cristae. Data are shown as mean  ±  SEM; * *p* < 0.001. ANOVA followed by Tukey post hoc multiple comparisons were used.

**Figure 6:**
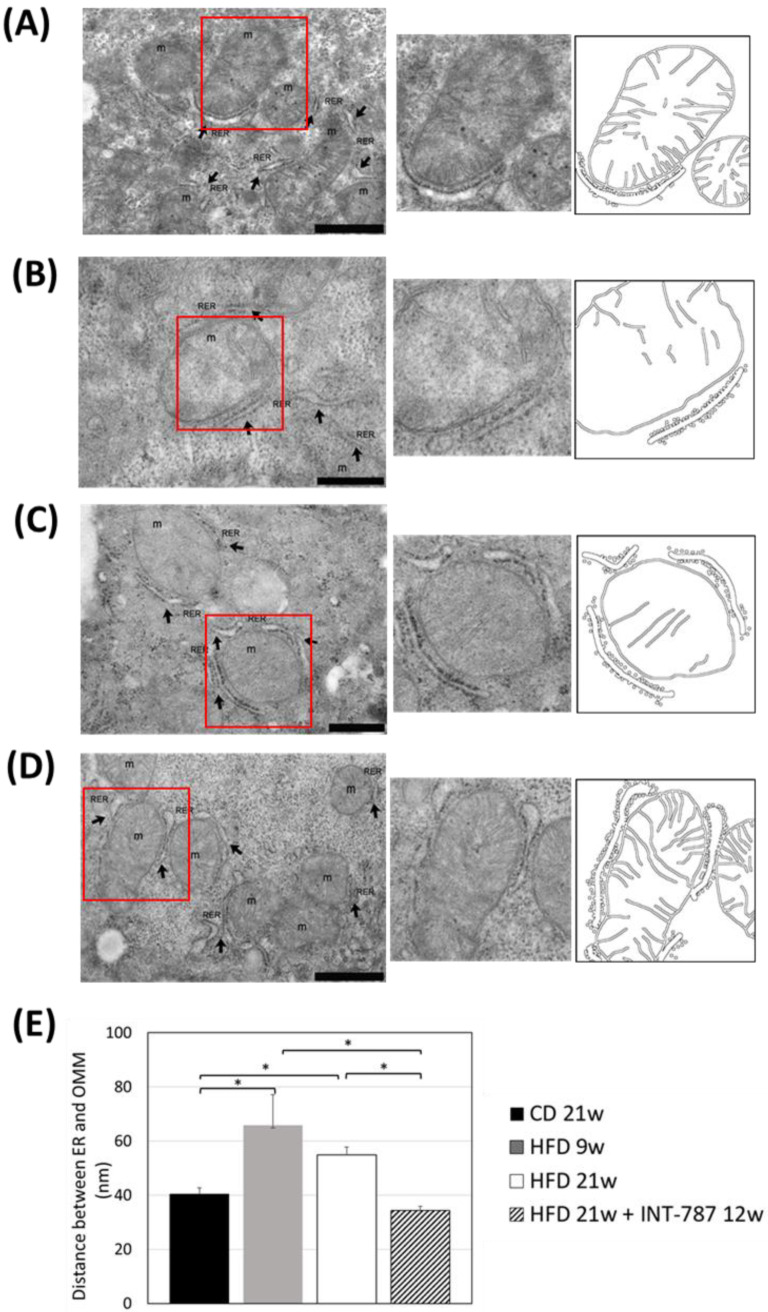
In vivo treatment with INT-787 restores the distance between ER and OMM in HFD-fed ob/ob mouse model of MASH. Representative TEM pictures: *n* = 3 independent experiments. Liver magnification: (**B**,**C**), bar 500 nm; (**A**,**D**), bar 1 µm; the selected area (red boxes) was subsequently enlarged (2× zoom), on the right, and analyzed using Image J (FIJI 1.54p). Legend: m: mitochondria, RER: rough endoplasmic reticulum, arrows: RER. (**E**) Image J quantification of the distance between ER and OMM (nm). Data are shown as mean  ±  SEM; * *p* < 0.0001. Kruskal–Wallis and Dunn’s tests were used.

The analysis by Image J (FIJI 1.54p) of TEM picture was performed to quantify ultrastructural changes in liver mitochondria. The images showed that HFD, after 9 and 21 wks, caused mitochondrial ultrastructure abnormalities, including a decrease in mitochondrial length (Figure 4) as well as a reduction in the number of CJs (Figure 5) and an increase in the distance between ER and OMM when compared with CD group (Figure 6). Mitochondria heterogeneous in shape and with abnormal cristae morphology and distribution were especially found after 21 wks HFD (Figure 4, Figure 5 and Figure 6).

After INT-787 administration, mitochondria showed normal features, with recovery of mitochondrial length (Figure 4), number of CJs (Figure 5) and distance between ER and OMM (Figure 6) as compared with HFD 9 and 21 wks. Notably, after INT-787 treatment, these markers were comparable with those found in the CD group (Figure 4, Figure 5 and Figure 6).

## 3. Discussion

The present study documents the recovery by INT-787 treatment of hepatic ATP, NADP(H) ratio and MIC19 mRNA which were downregulated after 21 wks of HFD. Reduced hepatic ROS formation and increased GSH levels have also been induced by INT-787 treatment of HFD-fed mice. Of note, INT-787 treatment also restored the mitochondrial length, the number of CJs and the distance between ER and OMM.

Recent evidence shows that mitochondria and ER play crucial roles in the pathogenesis and progression of MASLD [14,21]. In patients with MASH, mitochondria showed low maximal respiration, increased H_2_O_2_ production, lipid peroxidation, and decreased antioxidant capacity [6].

In the present study, we analyzed the impact of HFD feeding and the effect of INT-787 treatment on hepatic mitochondria morphology using a diet-induced ob/ob mouse model of MASH and in HFD 9 and 21 wks mice we found a reduction in mitochondrial length. Qualitative and quantitative assessment of TEM images showed smaller mitochondria in HFD mice when compared with elongated mitochondria in the CD group. Our data agree with those previously reported by Boland et al. (2018): fragmented mitochondria were found in ob/ob mice fed with the same diet used in the present study [22]. The authors also reported reduced mitochondrial function—assessed by coupling efficiency and proton leak—as well as decreased mtDNA levels, similar to findings in patients with MAFLD [23]. These alterations are believed to impair mitochondrial functions, including ATP synthesis and ROS production.

Of note, ROS may promote mtDNA mutation and degradation [24], leading to oxidative damage and liver injury, inflammation, and fibrosis. The increased ROS production is linked to hepatic damage, as reported in the present study using HFD ob/ob mice, as well as to inflammation and fibrosis, as we recently documented using the same MASH model [16]. The increase in ROS and reduction in GSH that we observed is consistent with recently published findings [25], using the same mouse experimental model and diet, and following a comparable timeline [25].

Here, INT-787 administration has been found to revert these events by restoring elongated mitochondria, increasing hepatic ATP content, reducing ROS formation and increasing GSH levels, as well as counteracting liver damage. INT-787 also increased liver NAD(P)H bound/free ratio assessed by autofluorescence (AF), previously reported to be correlated with ATP content [20]. In addition, as we recently documented, the beneficial effects of INT-787 include, beyond a marked inhibition of liver injury, also clear anti-inflammatory and antifibrotic effects [16].

The present study also demonstrates alterations in mitochondrial cristae and a significant reduction in the number of CJs in HDF mice, as well as a reduction in hepatic MIC19. Previous studies using MIC19 liver-specific knockout (LKO) mice showed the same dysfunctions [12].

MIC19 has been found to stabilize mitochondrial outer-membrane protein Sam50 (the key subunit of SAM complex) and inner-membrane protein Mic60 (the key component of MICOS complex) to form Sam50–Mic19–Mic60 axis, with a critical role for the maintenance of mitochondrial cristae architecture [26]. Recently, MIC19 has been reported to be cleaved by mitochondrial metalloprotease OMA1 in a postprandial mouse liver model [26]. Alterations in Sam50–Mic19–Mic60 axis have been found to cause changes in mitochondrial morphology, loss of mitochondrial cristae junctions, abnormal cristae formation and reduced ATP production [26]. Thus, the reduction in the number of CJs and the low mRNA MIC19 levels, here documented in HFD mice, explain the impaired ATP production.

In MASH, MIC19 expression, has been found downregulated [12] and restoring MIC19 expression could improve mitochondrial morphology and bioenergetics. It has been previously shown that INT-787 treatment in murine models leads to significant reductions in steatosis, lobular inflammation, and hepatocellular ballooning—components of the NAS score [16]. INT-787 reduced expression of fibrotic markers such as α-SMA and collagen I, and decreased Sirius Red staining in liver tissue, indicating attenuation of fibrosis progression. Improvements were also observed in ALT, AST, and bile acids, supporting systemic benefits aligned with clinical endpoints [16].

Additionally, the significant increase in serum ALT observed in MIC19 LKO mice [12] is in accordance with our data in HFD mice showing downregulated mRNA MIC19. The reversal effect of INT-787, able to increase mRNA MIC19 content, is associated with a complete recovery in liver damage, normalized ALT values, restored structure of mitochondrial cristae and increased number of CJs.

Recent evidence shows that MIC19 dysfunction not only induces abnormal mitochondrial structure, but is also linked to the development and progression of fatty liver disease [12]. MIC19 re-expression in MIC19 LKO mice has been found to restore liver lipid metabolism and to block liver diseases [12]. Additionally, in a methionine-choline deficient diet (MCD)-induced fatty liver model, MIC19 overexpression suppressed hepatic injury [12]. Interestingly, INT-787 treatment, able to counteract the mRNA MIC19 depletion, was found to protect the liver also against fatty accumulation by a reduction in number, area and diameter of hepatic lipid droplets [16].

Several studies have confirmed that ER stress, induced by diverse cellular stimuli, including redox imbalance, contributes to the pathogenesis of various liver diseases such as MASLD [27]. Notably, mitochondria-associated membranes (MAMs), the membranes representing the physical association between the ER and mitochondria, act as bridges for functional clustering of molecules, including lipids, and ROS exchange [14]. Previous studies have shown that MAM structural and functional integrity determines normal ER-mitochondria communication. During disruption of MAM integrity, a miscommunication causes imbalances in Ca^2+^ homeostasis and increases ER stress and oxidative stress [14]. Recently, using MIC19 LKO mice, an increase in the distance between ER and mitochondria, and mitochondrial membrane disorganization were reported [12]. The same events have also been documented in the present study using HFD mice. Notably, INT-787 administration fully restores the physiological distance between ER and mitochondria.

Previous evidence focused both on the FXR critical role in the regulation of fuel metabolism and energy homeostasis and its protective effect against tissue injury which resulted from the regulation of mitochondrial function and cellular turnover systems [28]. Recently, activation of FXR has been suggested to alleviate the Western diet-reduced OXPHOS, the process generating ATP in mitochondria [29].

Mitochondrial dysfunction exacerbates hepatic lipid accumulation, initiates inflammatory and fibrogenic responses, and induces cell death [2]. INT-787 treatment significantly restores the mitochondrial ultrastructure and reduces serum markers of liver injury, cholesterol, bile acids, and bilirubin.

A recent analysis of liver RNA sequencing data from human and mouse MASH samples has identified repressed genes, including those assigned to mitochondrial respiration-related pathways [30]. The authors demonstrated that the core pathogenetic factors in MASH patients include increased tissue injury, inflammation, and fibrogenesis, but also dysregulated energy metabolism and impaired mitochondrial function [30]. FXR agonists reverted the dysregulated MASH transcriptional network, as demonstrated by RNA sequencing using livers obtained from MASH mice [30].

In addition, previous studies in the liver have demonstrated that FXR loss facilitated the activation of the NLRP3 inflammasome and hepatocyte injury by ER stress and that these events were inhibited by the FXR agonist GW4065 [31].

Considering that a MIC19 decrease, leading to mitochondrial dysfunction [12,26], has been suggested to play a central role in MASLD and MASH [32], we could support the hypothesis of protective effects induced by INT-787 in HFD mice, demonstrating a recovery of mRNA MIC19 and a specific control on mitochondrial structures. INT-787 administration reverts changes in mitochondrial ultrastructure, as documented by restored mitochondrial morphology and mitochondrial cristae integrity, and protects against the decrease in mitochondrial length and number of CJs, as well as increasing the distance between ER and OMM. Although we specifically documented increased MIC19 mRNA levels induced by INT-787 treatment, with a similar trend observed in MIC19 protein levels, the link between FXR activation and mitochondrial homeostasis via MIC19 modulation needs to be further investigated.

The key question is whether INT-787 influences MIC19 expression directly—via FXR-mediated transcriptional regulation—or indirectly, by improving mitochondrial homeostasis. We hypothesize a potential direct regulation, as INT-787 treatment increases MIC19 mRNA levels; however, the precise regulatory pathway remains to be fully elucidated.

Furthermore, the ultrastructural and morphometric analyses employed to assess mitochondrial integrity reflect consequences rather than causes of mitochondrial dysfunction. Indeed, oxidative stress and disrupted energy production have been previously implicated as upstream contributors to mitochondrial dysfunction and liver damage [2].

Thus, our data support the accumulating evidence suggesting that specific interventions targeting mitochondrial homeostasis, including energy metabolism and mitochondrial quality control, represent promising strategies for MASH treatment [7,30].

A limitation of the present study is the use of a single mouse model, although this model is well validated for diet-induced MASH. Furthermore, the protocol employed was approved specifically for the collection of liver biopsies. As a result, it was not feasible to allocate liver tissue for mitochondrial isolation to enable direct analysis of mitochondrial respiration. This decision was made in accordance with the ethical principles of the 3Rs (Replacement, Reduction, and Refinement) that govern the use of animals in scientific research. To address this limitation, we plan to design a new experimental protocol specifically aimed at collecting liver tissue for mitochondrial isolation and functional analysis. Additionally, we acknowledge that the absence of direct MIC19 knockdown or overexpression experiments limits the strength of causal inference in the current study. These mechanistic investigations are part of our planned future work to further elucidate the role of MIC19 in FXR-mediated mitochondrial regulation.

## 4. Materials and Methods

### 4.1. Animal Model

Male B6V-LEP (ob/ob) mice, 6 wks-old, were obtained from Charles River Laboratories (Italy) and housed under standardized conditions (24 ± 2 °C; 12 h light–dark cycle). Mice were administered with HFD (AMLN diet-cod. D09100301) or with CD (cod. D09100304) by Laboratorio-Dottori-Piccioni (Italy). Mice were divided into 4 groups: (1) CD 21 wks (*n* = 7); (2) HFD 9 wks (*n* = 6); (3) HFD 21 wks (*n* = 10); (4) HFD 9 wks treated with HFD + INT-787 30 mg/kg for 12 wks (*n* = 10) (see Figure 1).

The dosing of INT-787 at 30 mg/kg/day was selected based on prior pharmacokinetic and pharmacodynamic studies conducted in rodent models, which demonstrated optimal FXR activation and therapeutic efficacy at this dose without signs of toxicity [17,33]. Additionally, dose–response data indicated that 30 mg/kg/day provided a robust pharmacological effect on hepatic lipid metabolism and fibrosis markers [33]. No significant differences in the reduction in plasma liver enzymes (ALT and AST), steatosis, or inflammation were observed among the tested doses of INT-787 (30, 60, or 120 mg/kg) [33]. Moreover, INT-787 is equipotent to OCA at FXR (EC50 = 0.14 μmol/L), and 30 mg/kg/day was also identified as the optimal dose for OCA [19].

All procedures involving animals were approved by the Italian Ministry of Health and by the University Commission for Animal Care (Protocol number 753/2020-PR; approved on: 10 May 2020). Baseline (9 wks) and terminal (21 wks) samples (liver and serum) collected in anesthetized mice (sodium pentobarbital 40 mg/kg i.p.) were snap-frozen in liquid nitrogen for further evaluations. We used the liver tissue biopsy for all the analysis and no mitochondrial and cytosolic fractions have been separated.

### 4.2. Real-Time qPCR

MIC19 mRNA expression was quantified by RT-qPCR. Total RNA was extracted from liver tissue homogenized in TRI reagent (Sigma-Aldrich, Milan, Italy), in accordance with the method described by Chomczynski et al. (1995) [34]. Total RNA concentration was determined by measuring the absorbance at 260 nm using a T92 + UV Spectrophotometer (PG Instruments Ltd., Lutterworth, UK), while purity was assessed using the 260/280 nm absorbance ratio. cDNA synthesis was synthesized using the iScript Supermix (BIO-RAD, Segrate, Milan, Italy). qPCR reactions were carried out on the CFX96^TM^ Real-Time System (BIO-RAD, Segrate, Milan, Italy) and by mixing 10 μL of SsoAdvancedTM SYBR^®^ Green Supermix (BIO-RAD, Segrate, Milan, Italy), 1 μL of the oligonucleotide forward/reverse primer mix (10 pmol/μL), and 2 μL of cDNA (2.5 ng/μL) to reach a final volume of 20 μL/well. Amplification was performed through two-step cycling (95–60 °C) for 40 cycles, following the supplier’s instructions. All samples were assayed in triplicate. Amplification efficiencies for *Mic19*, *Gapdh*, and *Rps9* were determined using standard curves (100.0%, 111.7%, 112.2%, respectively) in a cDNA concentration range of 10–0.625 ng/μL. The expression levels of the reference genes, *Gapdh,* and *Rps9*, were stable across all experimental conditions. The amplicon context sequence of the primers (PrimePCR, BIO-RAD, Segrate, Milan, Italy) *Mic19* (unique assay ID: qMmuCID0011070), *Gapdh* (unique assay ID: qMmuCED0027497) and *Rps9* (unique assay ID: qMmuCED0037603) are listed in Table 2, which lists the amplicon sequences with additional base pairs added to the beginning and/or end of the sequence. This adheres to the minimum information for the publication of real-time quantitative PCR experiments (MIQE) guidelines, as reported by Bustin et al. [35]. Gene expression was calculated by the ΔCt method and group comparison were performed using the ΔΔCt method.

### 4.3. Western Immunoblots

Liver tissues were homogenized in ice-cold lysis buffer supplemented with a protease inhibitor cocktail, followed by centrifugation at 15,000× *g* for 10 min. Equal amounts of protein from the resulting liver extracts were resolved by SDS-PAGE using 12% acrylamide gels and subsequently transferred onto PVDF membranes (Bio-Rad, Segrate, Milan, Italy). After blocking in 5% BSA in TBS Tween (20 mM Tris/HCl, 500 mM NaCl, pH 7.5, 0.1% Tween 20) for 2 h to avoid aspecific bonds, the membranes were incubated with primary antibodies (MIC-19: 1:1000, Abcam, Cambridge, UK; GAPDH: 1:20,000, Proteintech, Manchester, UK) overnight at 4 °C, under gentle agitation and then incubated with peroxidase-conjugated secondary anti-rabbit antibodies (Santa Cruz Biotechnology, Dallas, TX, USA). Immunostaining was revealed with BIO-RAD Chemidoc XRS+, visualized using the ECL Clarity (BIO-RAD), and intensity quantification was performed by Image Lab Software™ 6.0.1 (Bio-Rad).

### 4.4. Serum and Tissue Biochemistry

Serum ALT, ALP, total bilirubin, bile acids, cholesterol, and triglyceride were quantified by an automated Beckman Coulter AU 5820 (Brea, CA, USA) according to the manufacturer’s instructions.

ATP was measured using the luciferin–luciferase method with the ATPlite luminescence assay kit (Perkin Elmer Inc., Shelton, CT, USA), following the manufacturer’s instructions with some modifications. Briefly, frozen tissue was homogenized in ice-cold 0.1 M phosphate buffer containing 3 mM EDTA at pH 8.8 (50 mg/mL) and immediately precipitated with 30% trichloroacetic acid (TCA) in a 1:1 ratio. Samples were then centrifuged at 3000× *g* for 15 min. The supernatant was diluted in 100 mM phosphate buffer at pH 7.75, and luminescence was measured using a Perkin Elmer Wallac Victor2 multilabel plate reader in a white 96-well plate. ATP content was expressed per mg of protein.

Global assessment of oxidative stress, reactive oxygen species (ROS) generation, in liver biopsies was assessed by measuring the conversion of 2′,7′-dichlorofluorescein diacetate (H_2_DCF-DA) to fluorescent 2′,7′-dichlorofluorescein (DCF). Frozen liver biopsies were homogenized in ice-cold 0.1 M phosphate buffer containing 3 mM EDTA at pH 8.8 (100 mg/mL), and centrifuged at 1800× *g* for 10 min at 4 °C. The resulting supernatant was incubated with 5 µM H_2_DCF-DA for 10 min in the dark. Fluorescence was measured using a Perkin Elmer Wallac Victor2 multilabel plate reader (excitation/emission: 485/530 nm) in a black 96-well plate. Fluorescence intensity was expressed per mg of protein [36].

The hepatic concentration of glutathione (GSH) was measured by an enzymatic method following the manufacturer’s instructions (Cayman Chemical Co., Ann Arbor, MI, USA).

Protein content was determined by the method of Lowry et al. (1951) [37].

### 4.5. Transmission Electron Microscopy (TEM) Analysis

An effective protocol for accurate ultrastructural and morphometric analyses of cryo-stored bulk tissue samples was applied as published by Galhuber et al. (2021) [38]. Based on a comparative study with fresh tissue, the authors conclude that frozen tissue samples, appropriately treated, are suitable for ultrastructural morphology, thereby expanding the range of specimens available for retrospective ultrastructural investigations. Briefly, samples were cut into small pieces (~3 mm^3^) and fixed with 2.5% glutaraldehyde and 2% formaldehyde buffered in 0.1 M cacodylate buffer (pH 7.4) for 3 h at 37 °C. After rinsing in 0.1 M cacodylate buffer, the samples were post-fixed in 2% osmium tetroxide (OsO_4_) diluted in 0.2 M cacodylate buffer for 3 h at RT. Subsequently, specimens were rinsed in 0.2 M cacodylate buffer, dehydrated through a graded acetone series, and infiltrated in a 1:1 mixture of epoxy resin (EM-bed 812, Electron Microscopy Sciences, Hatfield, PA, USA) and pure acetone. Tissue samples were then embedded in gelatin capsules, and resin polymerization was performed at 60 °C for 48 h. Ultrathin sections (60–80 nm) were cut using a Reichert OM-U3 ultramicrotome (C. Reichert AG, Wien, Austria) and collected on nickel grids coated with a Formvar-carbon film (300 Mesh). The specimens were floated on 4% uranyl acetate and then briefly incubated in lead citrate as previously described [39]. Finally, the specimens were observed with a JEM 1200 EX II electron microscope (JEOL, Peabody, MA, USA) operating at 100 kV and equipped with a MegaView G2 CCD camera (Olympus OSIS, Tokyo, Japan).

A semiquantitative morphometric analysis of mitochondrial length and distance between ER and OMM has been performed on TEM image using Image-J software. In detail, before quantification, the software was calibrated using the scale bar from each TEM image (Figures S1–S8). 

The number of mitochondria, for each animal, analyzed for length quantification was as follows: CD 21 weeks (*n* = 8), HFD 9 weeks (*n* = 9) (C) HFD 21 weeks (*n* = 11) (D) HFD 21 weeks + INT-787 12 weeks (*n* = 10). The value was expressed in nm.

We have considered the ER–OMM distance to be the physical space between the ER and OMM at contact sites. The number of measures, for each animal, performed for the quantification of the distance between ER and OMM was as follows: CD 21 weeks (*n* = 11), HFD 9 weeks (*n* = 14), HFD 21 weeks (*n* = 13), HFD 21 weeks + INT-787 12 weeks (*n* = 12). The value was expressed in nm.

As cristae junctions (CJs), we measured the connections between inner boundary membrane (IBM) and the interior folds of the inner membrane known as cristae membranes. The number of CJs is expressed as the percentage of CJs per 100 mitochondrial cristae analyzed, as recently reported by Dong et al., 2024 [12].

Furthermore, the acquisition of electron microscope images was randomized and all quantifications (*n*  =  3 independent experiments) were performed in a blinded fashion by two independent investigators to minimize bias.

### 4.6. Autofluorescence Spectrofluorimetric Analysis

The hepatic autofluorescence (AF) of NADPH bound/free ratio was measured via fiber-optic probe by ex vivo spectrofluorometric and imaging analysis performed on liver tissue cryostatic sections. A significant correlation between liver NADP(H) bound/free and ATP value was previously reported [20]. Hepatic tissues sections cut at cryostat and collected on glass slides were submitted to AF microspectrofluorometric analysis. A curve fitting analysis was then applied to AF emission spectra to estimate the relative contribution of the signal of bound and free NAD(P)H and the subsequent calculation of the NADPH bound/free ratio values, according to a procedure previously reported [18]. Briefly, the AF emission spectra were measured using a multichannel analyzer (Hamamatzu PMA-12 photonic model, Hamamatsu Photonics Italia Srl, Arese, Italy), under fluorescence microscope operating in epi-illumination (Leica orthoplan model, Leica Microsystems S.r.l., Buccinasco, Milan, Italy) and a Leica 40 X objective (40 X Leica, n.a. 0.65). The excitation light (366 nm, from a LC-L1 UV-LED light source) was guided with a fiber optic probe to the excitation ingress of the microscope. The AF signals, in turn, were recorded in the 400–750 nm visible light interval through a 50/50 dichroic mirror and a 410 nm barrier filter. A curve-fitting procedure (PeakFit version 4; SPSS Science, Chicago, IL, USA), based on the Marquardt–Levenberg algorithm [40] and based on the use of half-Gaussian modified Gaussian functions describing the emission profile of the single endogenous fluorophore was then applied to estimate their contribution to the whole AF emission area. The relative contributions of NAD(P)H in the bound or free form estimated from each spectrum were then used to calculate the NAD(P)H bound/free ratios.

### 4.7. Statistical Analysis

Data are presented as means ± SEM. Differences among the four groups were estimated by one-way ANOVA followed by Tukey’s test for multiple comparisons. When data distribution was not normal, Kruskal–Wallis and Dunn’s tests were used. A *p* value < 0.05 was considered significant. Statistical analysis was performed using MedCalc Statistical Software (version 18.11.3).

## 5. Conclusions

In conclusion, our results underline the involvement of the FXR pathway in the control of mitochondrial damage, thus revealing a previously unknown mechanism mediated by FXR activation: upregulation of the IMM protein MIC19, essential for maintaining cristae integrity and mitochondrial function. INT-787 treatment significantly reduces serum markers of liver injury and hepatic ROS formation, reverts the decrease in GSH, ATP, and mRNA MIC19 levels and improves mitochondrial ultrastructure abnormalities, including the decrease in mitochondrial length, the loss of number of CJs and the increase in distance between ER and OMM. These data provide a novel starting point for basic research and drug development centered on mitochondria. Translating novel mitochondria-targeted approaches into effective and safe therapies will hopefully help to control hepatic diseases such as MASH.

## Figures and Tables

**Table 1:** Effects of INT-787 on liver weight and hepatic injury in a diet-induced ob/ob mouse model of MASH.

	Control	HFD 9 wks	HFD 21 wks	HFD 21 wks + INT-787 12 wks	Significance
**Liver weight (% BW)**	6.14 ± 0.67 ^#^	9.22 ± 0.22	9.49 ± 0.51	7.09 ± 0.14 ^#^	*p* ≤ 0.001
**ALT (mU/mL)**	349.17 ± 45.85	699.83 ± 36.53	534.55 ± 46.79	157.40 ± 152 ^$^	*p* ≤ 0.001
**ALP (mU/mL)**	213.67 ± 19.3	295.00 ± 19.5 ^$^	202.10 ± 9.44	204.20 ± 12.93	*p* ≤ 0.001
**Bilirubin (mg/dL)**	0.070 ± 0.019	0.100 ± 0.012	0.109 ± 0.015	0.040 ± 0.001 ^#^	*p* ≤ 0.002
**Bile Acids (µmol/L)**	24.54 ± 2.48	23.69 ± 5.79	21.92 ± 5.69	1.94 ± 0.08 ^$^	*p* ≤ 0.02
**Cholesterol (mg/dL)**	133.29 ± 18.43 ^#^	266.33 ± 21.45	252.34 ± 19.97	136.80 ± 6.17 ^#^	*p* ≤ 0.001
**Tryglicerides (mg/dL)**	84.57 ± 6.04	78.67 ± 4.99	80.00 ± 5.67	73.90 ± 5.99	ns

Data are shown as mean  ±  SEM; ^$^ vs all group; ^#^ vs HFD 9 and 21 wks; ns: no significance. Mice groups: CD 21 wks (*n* = 7), HFD 9 wks (*n* = 6), HFD 21 wks (*n* = 10) and HFD 21 wks treated with INT-787 30 mg/kg for 12 wks (*n* = 10). ANOVA followed by Tukey post hoc multiple comparisons were used for the analysis of liver weight, ALT, ALP, and cholesterol. When data distribution was not normal, Kruskal–Wallis and Dunn’s tests were used: bilirubin, bile acids and tryglicerides.

**Table 2:** Amplicon sequences of primers. *Mic19* or so called coiled-coil-helix-coiled-coil-helix domain containing 3 (*Chchd3*) was the gene of interest to be analyzed. Glyceraldehyde-3-phosphate dehydrogenase (*Gapdh*), and ribosomal protein S9 (*Rps9*) were used as reference genes.

Gene	Amplicon Sequence
*Chchd3/Mic19* (ID: qMmuCID0011070)	TCAGCCTGTATAAGGAAGTGACTGACTCCAGGACAGCACAGATCATGTTTCAGG CGTATGGAGACAAGCAGGGACCACTGCATGGAATGTTAATCAATACTCCGTATG TGACCAAAGACCTTCTTCAATCAAAGAGGTTTCAG
*Gapdh* (ID: qMmuCED0027497)	TGGGAGTTGCTGTTGAAGTCGCAGGAGACAACCTGGTCCTCAGTGTAGCCCAAG ATGCCCTTCAGTGGGCCCTCAGATGCCTGCTTCACCACCTTCTTGATGTCA
*Rps9* (ID: qMmuCED0037603)	CGTCCAGGCCGAGTGAAGAGGAAGAATGCCAAGAAAGGCCAGGGCGGGGCTG GAGCTGGTGATGATGAGGAAGAGGATTAATTAATACTTGGCTGAACTGGAGGAT TGTCTAGTTTTCC

## Data Availability

The data presented in this study are available on request from the corresponding author.

## Supplementary Materials

The following supporting information can be downloaded at: https://www.mdpi.com/article/10.3390/ijms262211023/s1.

## Abbreviations

The following abbreviations are used in this manuscript:

CJs	Cristae Junctions (CJs)
ER	Endoplasmic Reticulum
IBM	Inner Boundary Membrane
IMM	Inner Mitochondrial Membrane
OMM	Outer Mitochondrial Membrane
