# Supplementary material for: Farnesoid X Receptor Agonist INT-787 Inhibits Hepatic Mitochondrial Dysfunction in a Diet-Induced ob/ob Mouse Model of MASH
Source: Int J Mol Sci. 2025 Nov 14;26(22):11023. doi: 10.3390/ijms262211023 (PMC12652301; doi:10.3390/ijms262211023)
Supplement: Supplementary file 1 [file ijms-26-11023-s001.zip › ijms-3895448-supplementary.pdf]

Farnesoid X receptor agonist INT-787 inhibits hepatic mitochondrial dysfunction in a diet-induced ob/ob mouse model of MASH

Laura G. Di Pasqua, Giuseppina Palladini, Anna C. Croce, Gloria Milanesi, Margherita Cavallo, Luciano Adorini, Andrea Ferrigno, Mariapia Vairetti

Representative TEM images for each experimental group, analyzed using Image J, are reported in Figures S1–S8. Hepatic mitochondrial length and the distance between the endoplasmic reticulum (ER) and the outer mitochondrial membrane (OMM) were evaluated.

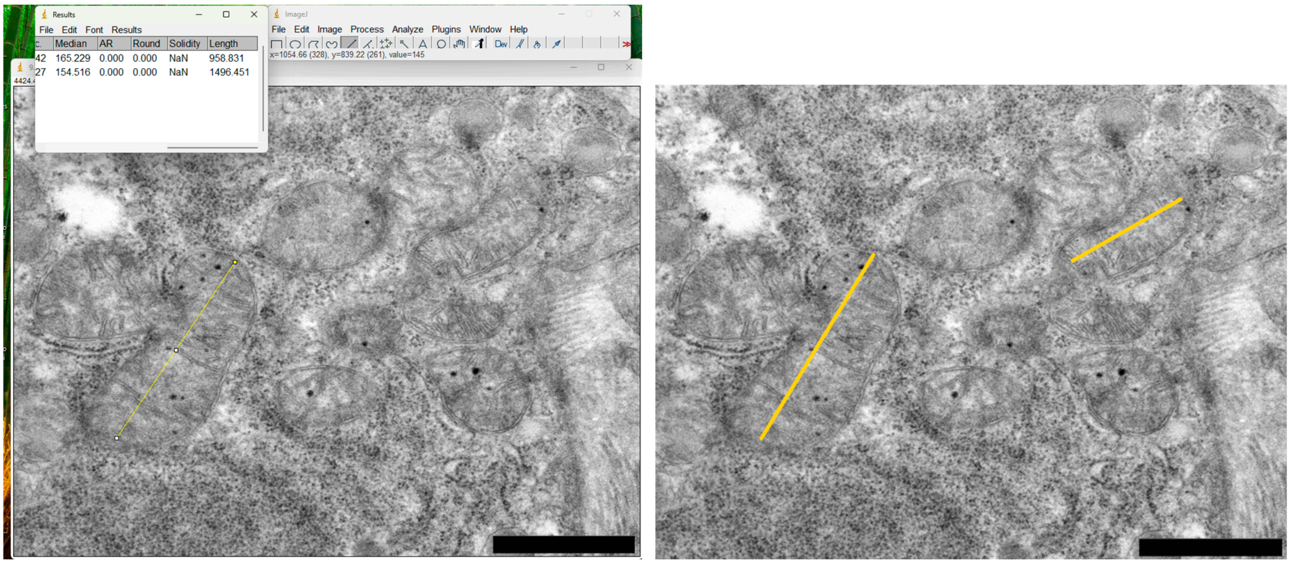

Figure S1. Representative TEM pictures showing the evaluation of hepatic mitochondrial length in CD 21 wks. Liver magnification: bar 1 µm. For greater clarity, two images of the same sample are shown. On the left is the screenshot of the analysis conducted with Image J; on the right is the same image with the measurements indicated, since the Image J software does not allow displaying multiple evaluations simultaneously, but only one at a time.

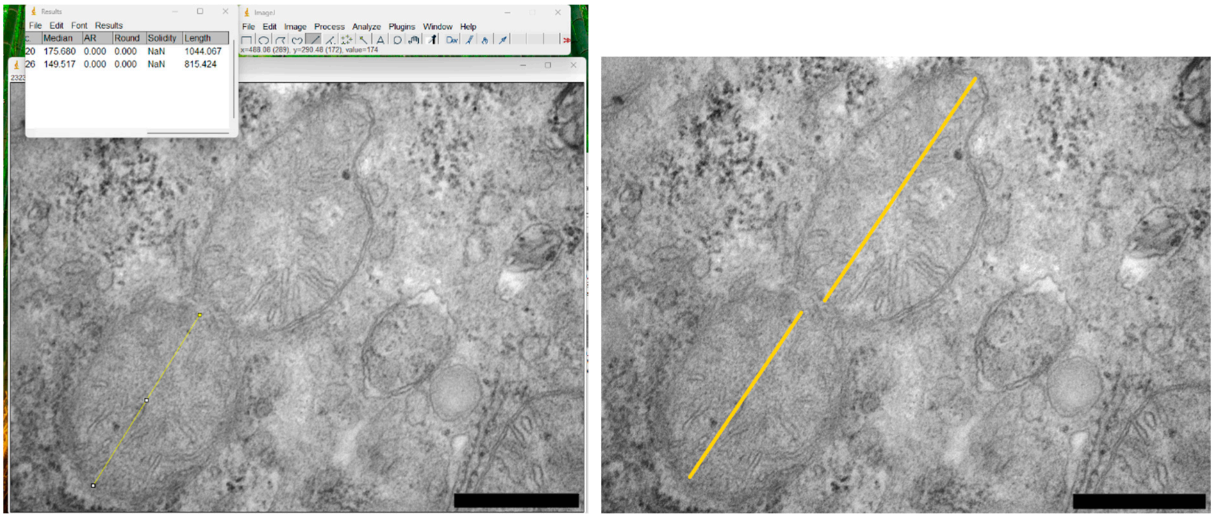

Figure S2. Representative TEM pictures showing the evaluation of hepatic mitochondrial length in HFD 9 wks. Liver magnification: bar 500 nm. For greater clarity, two images of the same sample are shown. On the left is the screenshot of the analysis conducted with Image J; on the right is the same image with the measurements indicated, since the Image J software does not allow displaying multiple evaluations simultaneously, but only one at a time.

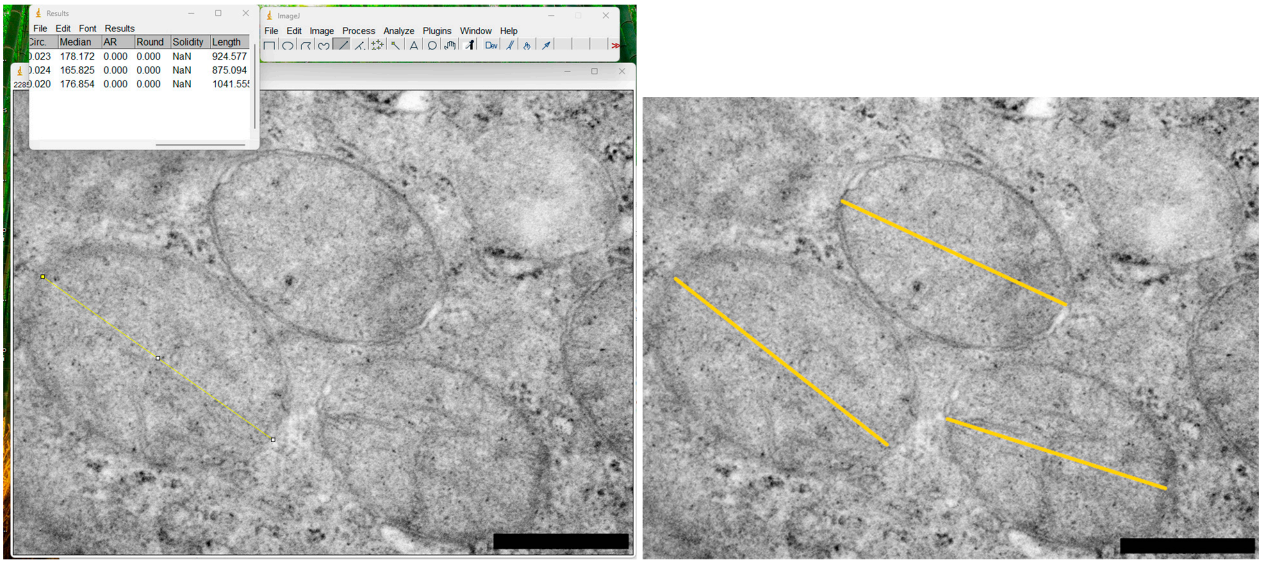

Figure S3. Representative TEM pictures showing the evaluation of hepatic mitochondrial length in HFD 21 wks. Liver magnification: bar 500 nm. For greater clarity, two images of the same sample are shown. On the left is the screenshot of the analysis conducted with Image J; on the right is the same image with the measurements indicated, since the Image J software does not allow displaying multiple evaluations simultaneously, but only one at a time.

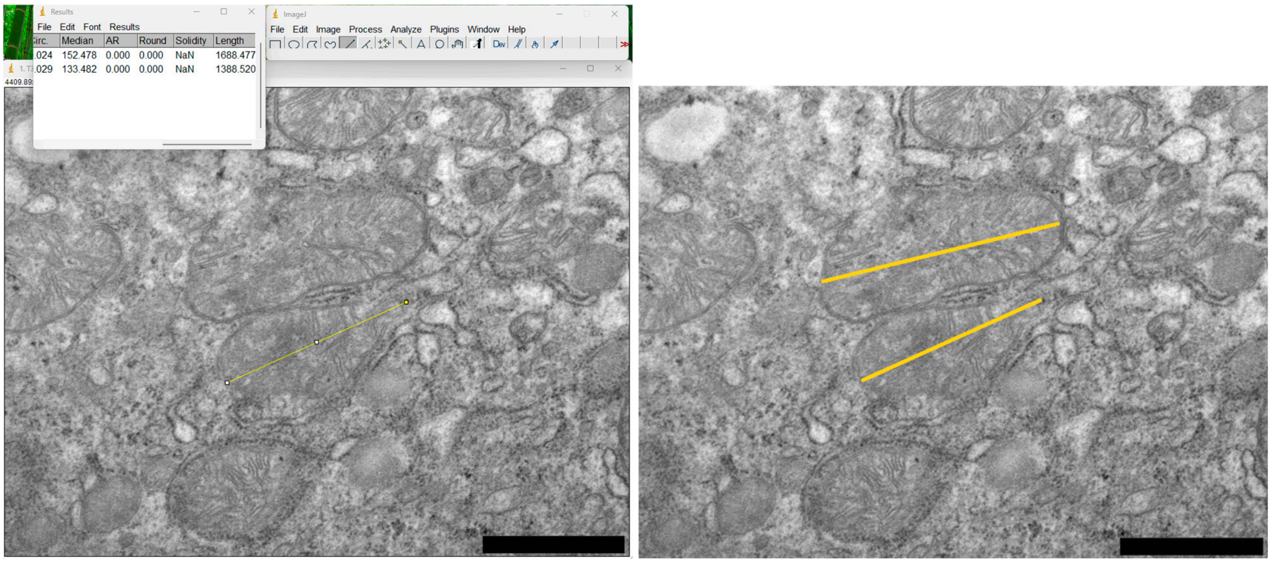

Figure S4. Representative TEM pictures showing the evaluation of hepatic mitochondrial length in HFD 21 wks. Liver magnification: bar 1 µm. For greater clarity, two images of the same sample are shown. On the left is the screenshot of the analysis conducted with Image J; on the right is the same image with the measurements indicated, since the Image J software does not allow displaying multiple evaluations simultaneously, but only one at a time.

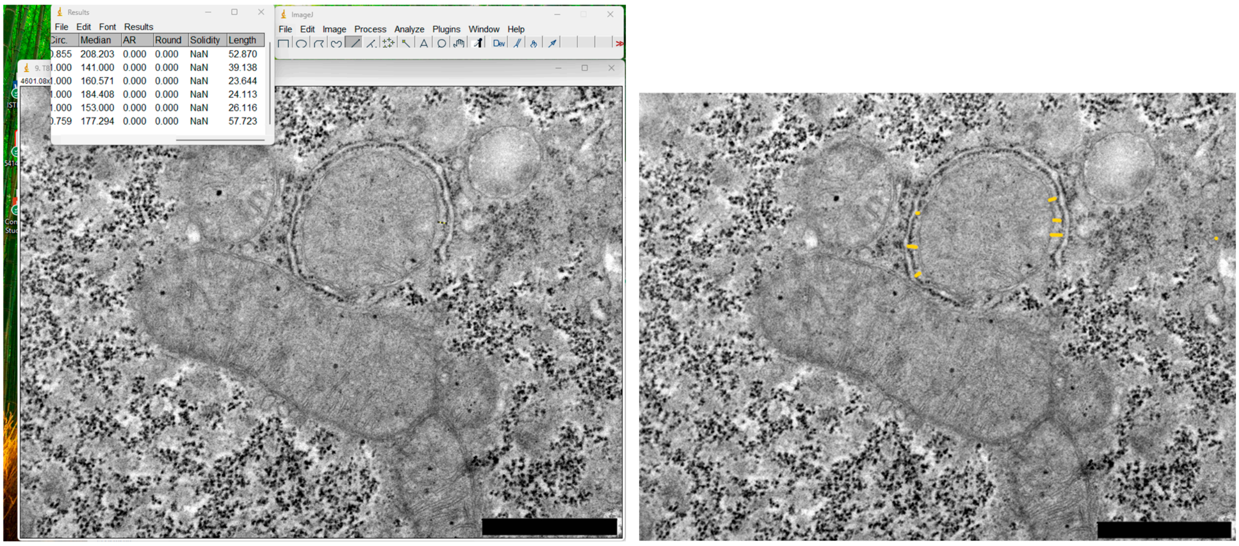

Figure S5. Representative TEM images showing the evaluation of the distance between ER and OMM in CD 21 wks. Liver magnification: bar 1 µm. For greater clarity, two images of the same sample are shown. On the left is the screenshot of the analysis conducted with Image J; on the right is the same image with the measurements indicated, since the Image J software does not allow displaying multiple evaluations simultaneously, but only one at a time.

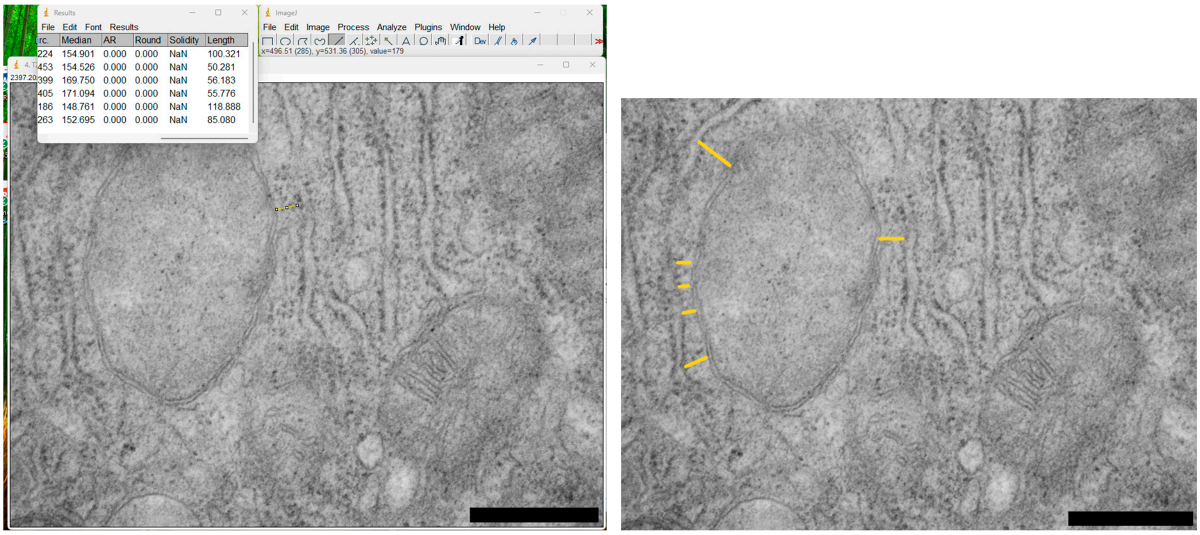

Figure S6. Representative TEM images showing the evaluation of the distance between ER and OMM in HFD 9 wks. Liver magnification: bar 500 nm. For greater clarity, two images of the same sample are shown. On the left is the screenshot of the analysis conducted with Image J; on the right is the same image with the measurements indicated, since the Image J software does not allow displaying multiple evaluations simultaneously, but only one at a time.

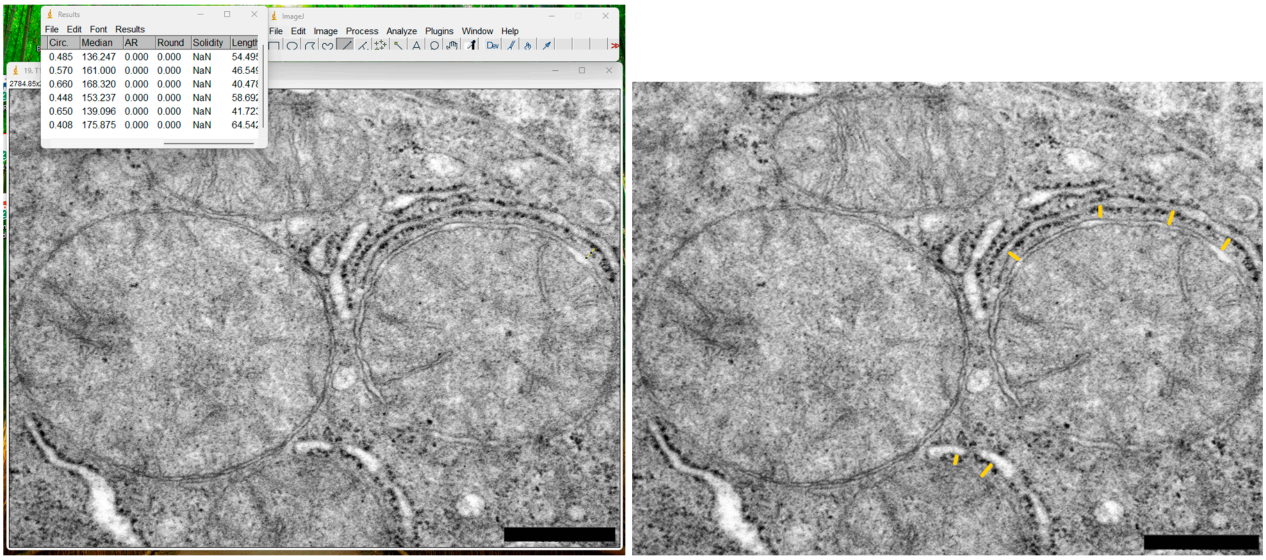

Figure S7. Representative TEM images showing the evaluation of the distance between ER and OMM in HFD 21 wks. Liver magnification: bar 500 nm. For greater clarity, two images of the same sample are shown. On the left is the screenshot of the analysis conducted with Image J; on the right is the same image with the measurements indicated, since the Image J software does not allow displaying multiple evaluations simultaneously, but only one at a time.

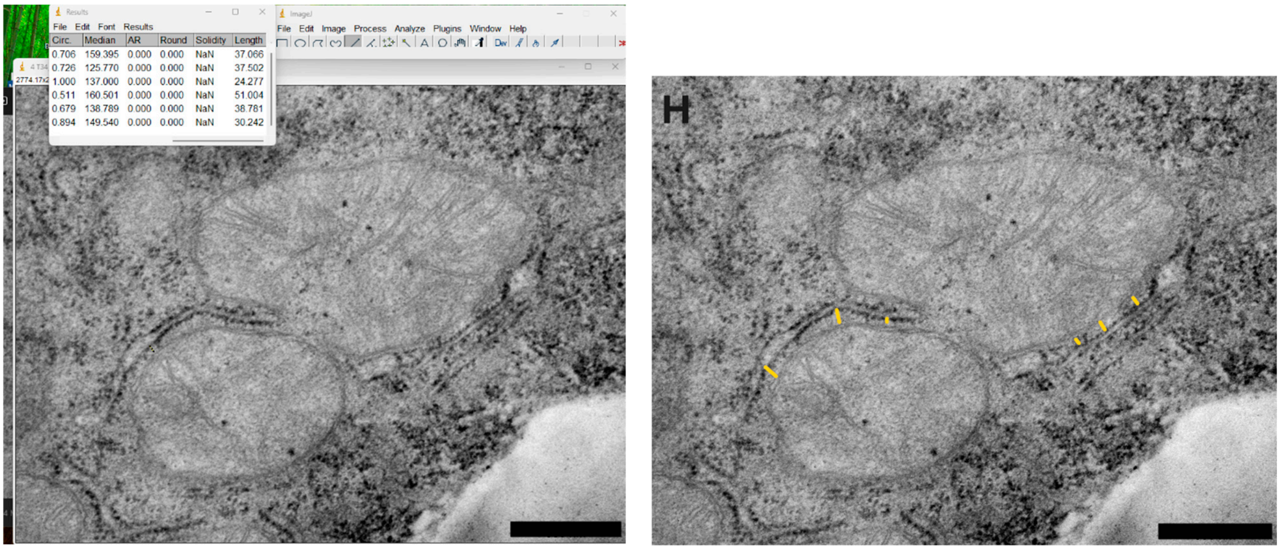

Figure S8. Representative TEM images showing the evaluation of the distance between ER and OMM in HFD 21 wks treated with INT-787 12wks. Liver magnification: bar 500 nm. For greater clarity, two images of the same sample are shown. On the left is the screenshot of the analysis conducted with Image J; on the right is the same image with the measurements indicated, since the Image J software does not allow displaying multiple evaluations simultaneously, but only one at a time.
